# Differences in the effect of adolescents’ strategies for expressing academic emotions on academic emotions and peer acceptance in competitive and cooperative situations

**DOI:** 10.3389/fpsyg.2024.1407885

**Published:** 2024-07-02

**Authors:** Ying Liu, Xiaoyun Chai, Biao Sang, Shaohua Zhang

**Affiliations:** ^1^Wenbo College, East China University of Political Science and Law, Shanghai, China; ^2^Department of Applied Psychology, Hubei University of Medicine, Shiyan, China; ^3^Lab for Educational Big Data and Policymaking, Shanghai Academy of Educational Sciences, Shanghai, China; ^4^School of Psychology and Cognitive Science, East China Normal University, Shanghai, China; ^5^School of Preschool Education, Xi’an University, Xi’an, China

**Keywords:** adolescents, academic emotion, emotional suppression, emotional expression, competitive situation, cooperative situation

## Abstract

Two studies were conducted to explore the differences in the effect of adolescents’ strategies for expressing academic emotions. In Study 1 a total of 65 adolescents participated in the study of the relationship between academic emotions and strategies for expressing them in competitive and cooperative situations. In Study 2 a total of 113 adolescents participated in the study of the relationship between the strategies and peer acceptance in competitive and cooperative situations. The results showed that the relationship between academic emotions and strategies for expressing them in competitive and cooperative situations was situation stable while the relationship between the strategies and peer acceptance was situation specific. Furthermore, emotional expression may be more adaptive when experiencing positive academic emotions. When adolescents experience negative academic emotions, expressing them is more adaptive from the perspective of their own academic emotional experience; whereas suppressing them is more adaptive from the perspective of peer acceptance. These findings (a) clarify how to use more adaptive strategies for emotional expression in various situations and (b) serve as a guide for helping adolescents use strategies to express emotions flexibly according to the situation.

## 1 Introduction

Academic emotions refer to various emotional experiences (e.g., happy, pride, anxiety) associated with students’ academic achievements in the course of instruction or studying (Pekrun et al., 2002). Previous researchers have found that academic emotions have a positive or negative impact on learning processes and learning outcomes (Rodríguez-Muoz et al., 2021; Wang et al., 2022). Furthermore, these emotions may even have long-term effects on individuals’ relationships and mental health (Rojas et al., 2014; McLaughlin et al., 2015). To improve the beneficial role of all kinds of academic emotions, researchers began to focus on emotional regulation in academic settings (Ben-Eliyahu and Linnenbrink-Garcia, 2013). This skill impacts not only an individual’s ability to interpersonal communication, collaboration, and decision-making, but also their success in academic and professional fields (Warrier et al., 2021; Tripon, 2023). In the domain of emotional regulation, strategies for expressing emotions include (a) emotional suppression, or the conscious inhibition of one’s own emotionally expressive behavior while emotionally aroused; and (b) emotional expression, or the conscious revelation of one’s emotions while emotionally aroused (Gross, 1989, 1998). These strategies can be applied to emotions experienced in academic situations.

Previous studies have confirmed that strategies for expressing emotions not only have a social function but can also have unique effects on internal (e.g., emotions) and interpersonal (e.g., peer relationships) interactions (Cameron and Overall, 2018; Liu et al., 2019). Early researchers, however, rarely focused on strategies for expressing academic emotions and also ignored the influence of the situation to some extent. According to the functional orientation of emotion regulation, the situation is particularly important in the process of that regulation; people must match their emotional expression with the situation (Greenaway and Kalokerinos, 2017). The situation is likely to prove vital to understanding the effects of strategies for emotional expression, given that emotional suppression and expression are often used to communicate individuals’ perceptions of the situation and intention toward others (Chavez-Baldini et al., 2020; Manokara et al., 2022). Accordingly, the primary aim of the current study was to examine the effects of strategies for expressing academic emotions on Chinese adolescents in competitive and cooperative situations, focusing on the relationship between academic emotions and the strategies for expressing them as well as the relationship between the strategies and peer acceptance.

### 1.1 Effects of strategies for expressing academic emotions

Research on the effects of strategies for expressing emotions has focused primarily on two aspects. The first is the relationship between the strategies and individual emotional experience. Specifically, in the academic setting, results from several studies have indicated that the expression of emotions is generally regarded as an effective strategy for their regulation (Rimé, 2007; Yan, 2012). To a large extent, however, the suppression of emotions is an inappropriate strategy in learning because it can weaken positive academic emotions and enhance negative ones (Liu et al., 2019; Somerville and Whitebread, 2019). The second is the relationship between strategies for expressing emotions and social outcomes. Research on this relationship in the academic field is relatively lacking, but studies from a holistic standpoint have shown that the habitual suppression of emotional expression may impair social function (e.g., peer relationship quality) by blocking information exchange and expanding interpersonal distance from peers (Tsai et al., 2017; Cameron and Overall, 2018; Chervonsky and Hunt, 2018). On the contrary, individuals can convey friendliness and motivation to establish close relationships by expressing positive emotions (Greenaway and Kalokerinos, 2017); compared with individuals who express less positive emotion, these individuals were also perceived as more popular (Reysen, 2005) and more sociable (Pollastri et al., 2018).

In general, most previous researchers have tended to regard the suppression of emotions as a nonadaptive strategy, and the expression of emotion, especially positive emotions, as an adaptive strategy; however, they have generally ignored the important role of the situation.

### 1.2 Effect of strategies for expressing academic emotions: role of the situation

The reason that the suppression and expression of emotion cannot be easily regarded as fully “adaptive” or “maladaptive” is that the situation may play a role in the effectiveness of the two strategies (Greenaway and Kalokerinos, 2017; Chavez-Baldini et al., 2020; Paul et al., 2023). The process model of emotion regulation proposed by Gross (2015) not only proposes that the entire process of emotion regulation contains five types of emotion regulation strategies: situation selection, situation modification, attentional deployment, cognitive change and response modulation, but also puts forward the view that the effect of emotion regulation is jointly affected by strategy and situation, emphasizing the flexibility of emotion regulation. Emotional regulation flexibility refers to the ability of individuals to flexibly deploy and adjust regulation strategies according to the needs of different situations and the characteristics of regulation strategies to achieve the optimal regulation effects (Bonanno and Burton, 2013; Aldao et al., 2015; Pruessner et al., 2020). The exploration of expression flexibility is an important aspect of the study of emotional regulation flexibility, that is, the flexible transformation of both emotional expression and suppression strategies according to the changing requirements of the situation (Bonanno et al., 2004; Westphal et al., 2010). In other words, individuals need to flexibly choose strategies for expressing emotion that match the goal of emotion regulation in the appropriate situations. If the goal of an individual’s emotional regulation in a certain situation is to maintain harmonious interpersonal relationships, then a strategy that can promote good relationships should be chosen, rather than only considering their own enjoyment. Only when the goal and strategy of emotion regulation can be flexibly selected according to the requirements of the situation can there be a better effect of emotion regulation. Thus, it can be seen that which of the two strategies of emotion expression and suppression has the better effect is no longer the focus of debate, but the flexibility of choosing and using both strategies based on situations is crucial (Aldao et al., 2015; Haines et al., 2016).

Furthermore, the theories of self-regulated learning suggest that emotional regulation is also an important component in self-regulated learning, and even at the core of the self-regulated learning process (Zimmerman, 2001; Pekrun et al., 2002; Boekaerts, 2007). In learning situations, self-regulated learners use emotion regulation strategies to manage the emotions they face, and the importance of these strategies lies in regulating emotions and emotional responses that may disrupt learning in order to achieve set learning goals (Schunk and Zimmerman, 1994) and cope with challenging situations or others in situations (Corno and Kanfer, 1993). At the same time, the theories of self-regulated learning also emphasize that these emotion regulation strategies are also situational, and different strategies have different adaptability in different learning situations (Ben-Eliyahu and Linnenbrink-Garcia, 2013; García-Pérez et al., 2021).

Many studies have also confirmed these theoretical viewpoints. Related studies in academic contexts, especially unpleasant situations, have shown that an inordinate expression of emotions is usually inappropriate; but even hiding the signs of unpleasant and inappropriate emotions from others can be seen as adaptive in academic situations (Burić et al., 2016). Some researchers have also found that the suppression of emotion was negatively related to college students’ positive emotions in favorite classes but positively related to their positive emotions in least favorite classes (Ben-Eliyahu and Linnenbrink-Garcia, 2013). Moreover, the positive social effects of expressing positive emotions cannot be generalized across all situations. For example, expressing one’s joy in the presence of classmates who performed poorly may not be appropriate. Compared with expressing positive emotions, outperformers may preferentially suppress the expression of positive emotions (Gross and Levenson, 1993) to obtain social benefits (Schall et al., 2016). These findings not only show that the effect of strategies for expressing academic emotion on the emotional experience and social outcomes may be situational, but also emphasize the need to investigate the situational specificity of that effect. We will next explore this topic by applying these two broad emotion regulation strategies to more specific learning situations.

### 1.3 Effect of strategies for expressing academic emotion: differences in competitive and cooperative situations

Adolescents spend much of their time in school (Seligman et al., 2009), which is a social subsystem full of competition and cooperation (Zhang, 2008; Li, 2017). At school, adolescents compete for first place in the exam while also cooperating for a good place in the learning group. Therefore, situations that adolescents experience generally manifest as competitive or cooperative (Chan and Lam, 2008; Torrego-Seijo et al., 2021). Social interdependence theory holds that the structure of goals that people pursue in a given social situation determines how they interact, and how they interact in turn largely determines the outcome of that situation (Johnson and Johnson, 1998). In the competitive situation characterized by negative interdependence, the individual goals within the group are reflected as “exclusive interdependence.” Individuals consciously want to win or perform better than others, focusing on improving the likelihood of achieving their own goals. Therefore, individuals fight with each other, which not only affects their learning behavior, but also leads to more negative interpersonal relations and mental health problems (Johnson and Johnson, 1989). In cooperative situations characterized by positive interdependence, individuals are closely related to the goals of others, showing a relationship of “facilitative interdependence.” Therefore, individuals are more likely to interact with each other through mutual help, exchange of resources, effective communication and other ways to achieve common goals, resulting in more active learning behaviors, harmonious interpersonal relationships and good mental health. Researchers have compared individual behavior in competitive and cooperative situations. For example, the results of the meta-analysis study found that individuals achieved higher achievement in cooperative situations than in competitive situations (Johnson and Johnson, 1989). Studies involving adolescents also found that individuals in cooperative situations spend more time on tasks and have a more positive attitude than those in competitive situations (Wentzel, 1989). However, there is a lack of research on the influence of adolescents’ strategies for expressing academic emotion on their emotional experience and social outcomes in competitive and cooperative situations. Further investigation of this issue is still of considerable theoretical significance and practical value.

#### 1.3.1 Relationship between academic emotions and strategies for expressing them: differences between competitive and cooperative situations

Previous studies on the relationship between academic emotions and strategies for expressing them showed that expressing emotion may enhance the positive emotional experience while suppressing emotion may lead to the reduction of positive academic emotions and the enhancement of negative academic emotions (Yan, 2012; Burić et al., 2016). These relationships, however, require further exploration in the context of competition and cooperation.

#### 1.3.2 Relationship between strategies for expressing academic emotion and peer acceptance: differences between competitive and cooperative situations

As for the relationship between strategies for expressing academic emotion and social outcomes, in this study we focused on the degree of acceptance of others when they use those strategies. Peer acceptance as the degree to which an individual is liked or accepted by other members of the peer group (Parker and Asher, 1993), is closely related to adolescents’ physical and mental health (Lam et al., 2021; Aguilar-Pardo et al., 2022) and academic achievement (Wentzel et al., 2021). During adolescence the importance of parents decreases, and peers become increasingly important (Macek and Ježek, 2007). When adolescents are in school, they spend most of their time with their peers, hoping to maintain their social status by interacting with their peers (Zettergren, 2007). In order to promote positive interaction with peers, adolescents need to develop and flexibly use a variety of emotional regulation strategies to gain a high degree of peer recognition (Wang et al., 2020).

Studies on the relationship between strategies for expressing academic emotion and peer acceptance in competitive and cooperative contexts are scarce. Similar studies, however, have shown that compared with the confrontational relationship in the competitive situation, the relationship among group members in the cooperative situation presents a mode of positive interdependence (Liu and Wang, 2005), so adolescents may experience out-group and in-group membership in competitive and cooperative situations, respectively, which may be important clues to determine whether suppressing or expressing positive emotions is appropriate (Paulus et al., 2016; Greenaway and Kalokerinos, 2017). In addition, the implied affiliative goal of positive emotional expression is matched with cooperative situations defined by shared goals and interdependence but mismatched with the presumed focus on the personal gain inherent in competitive situations. Expressing positive emotions in cooperative situations may, therefore, be beneficial to social interaction (Van Kleef, 2010). For example, Heerdink et al. (2013) found that group members who express happiness in cooperative situations put other group members at ease and reduce the pressure to conform compared to competitive situations. De Melo et al. (2014) asked participants to play a prisoner’s dilemma game and found that in a cooperative situation they were more willing to continue cooperating with a virtual partner who expressed joy. In general, however, the relationship between strategies for expressing academic emotion and peer acceptance in competitive and cooperative situations requires further exploration.

Overall, the goal of the current study was to explore the impact of adolescents’ strategies for expressing academic emotion on their own academic emotional experience and on peer acceptance in competitive and cooperative situations. Therefore, the current study comprised two substudies. In Study 1 we sought to examine the situational differences in the associations between academic emotion and the strategies for expressing them. Based on relevant theories and previous research in academic settings, specific hypotheses for Study 1 were proposed: (a) In competitive and cooperative situations, emotional expression would enhance the positive emotional experience; and (b) emotional suppression would reduce positive academic emotions and enhance negative academic emotions. The purpose of Study 2 was to explore the situational differences between those strategies and peer acceptance. We proposed that (a) in cooperative situations, expressing positive emotions would garner a higher level of peer acceptance; and (b) given the lack of relevant studies, we did not put forward any other specific hypotheses.

## 2 Study 1

### 2.1 Method

#### 2.1.1 Participants

Participants were 65 adolescents randomly recruited from two public, regular middle-high schools that located in Shandong province in China. All adolescents had normal or corrected vision, with no color blindness or color weakness. Thirty-one adolescents (age range = 12–18 years; *M*_age_ = 15.39 years, *SD* = 1.78 years; 15 boys) were randomly assigned to the competitive situation. Thirty-four adolescents (age range = 13–18 years; *M*_age_ = 15.71 years, *SD* = 1.59 years; 24 boys) were randomly assigned to the cooperative situation. The Chi-square test showed no significant difference in gender and grade between the two situations (*χ*^2^ = 3.33, *p* = 0.07 > 0.05, φ = 0.23; *χ*^2^ = 0.42, *p* = 0.52 > 0.05, φ = 0.08). The results of the independent sample *t*-test also showed no significant difference in the age of the subjects under the two situations, *t* (63) = −0.76, *p* = 0.45 > 0.05, Cohen’s *d* = 0.19. Sensitivity analyses indicated our sample size to be sufficiently powered at 80% for detecting small effects in regression models (Cohen’s *f* = 0.18).

#### 2.1.2 Experimental design

We used a mixed experimental design in the current study with (a) the situation (competition vs. cooperation) as the within-subjects factor, (b) both academic emotion (positive vs. negative) and regulation strategy (suppression vs. expression) as the between-subjects factor, and (c) academic emotion as the dependent variable.

#### 2.1.3 Experimental material

Given that emotional images are not effective in inducing students’ academic emotions, Takahashi et al. (2008) used short sentences (e.g., “I got a perfect score in mathematics”) to evoke feelings of pride and joy in students. Therefore, in Study 1 we used stories of academic emotion instead of images to induce adolescents’ academic emotions. The specific process of compiling and screening the stories follows: First, through an open questionnaire, 32 positive and 32 negative stories of academic emotion were selected for the competitive situation and 19 positive and 19 negative stories for the cooperative situation. Then, 55 adolescents (*M*_age_ = 12.27 years, *SD* = 1.82 years; 34 boys) were randomly selected to rate (a) emotional valence on a 9-point scale, ranging from 1 (*not at all happy*) to 9 (*very happy*), and (b) the arousal they experienced on a 9-point scale, ranging from 1 (*not at all strong*) to 9 (*very strong*). Finally, in the competitive situation, 15 positive (e.g., “In Chinese writing lessons, the teacher thought my composition was well written and not only commended me, but also read my compositions aloud to all of my classmates as examples”) and 15 negative stories of academic emotion (e.g., “In math class, the teacher let me do the mathematical problems written on the blackboard, but I did incorrectly, which made me feel like I was back on the seat in the sight of my classmates”) were selected (*M*_positive valence_ = 7.22, *M*_positive arousal_ = 7.05, *M*_negative valence_ = 2.04, *M*_negative arousal_ = 4.74, rated on a 9-point scale). In the cooperative situation, 15 positive (e.g., “We collaborate to think through and come up with solutions to the math problems that the teacher assigns, and we ultimately receive the whole grade”) and 15 negative stories of academic emotion (e.g., “Last week, our group ranked last in class and was punished by teachers due to the poor performance of our group’s classmates in all aspects”) were selected (*M*_positive valence_ = 7.03, *M*_positive arousal_ = 6.93, *M*_negative valence_ = 2.88, *M*_negative arousal_ = 4.47).

#### 2.1.4 Experimental procedure

**Figure 1:**
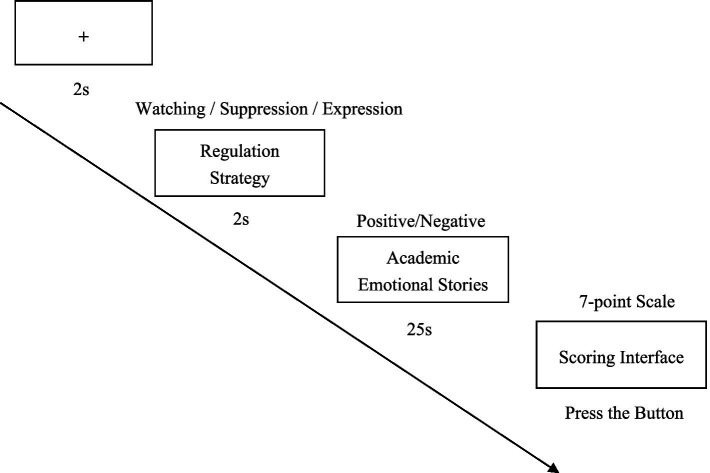
The flow of each trial.

Study 1 was approved by the institutional review board at our university. In this study emotional images were replaced with stories of academic emotion, using an adapted Reactivity and Regulation-Image Task (Carthy et al., 2010). The entire experiment was programmed with E-prime 2.0. The flow of each trial appears in Figure 1. A “+” is displayed on the first screen for 2 s, at which point the participants start to pay attention. The second screen displayed the participants’ strategy for expressing academic emotions (emotional suppression, emotional expression, or watching) for 2 s at a time. The stories of academic emotion will be shown on the third screen and run for 25 s. During this procedure, participants will employ the strategy provided on the second screen to regulate the academic emotion induced by the stories of academic emotion. A rating screen is shown to participants on the fourth screen, asking them to rate their positive or negative academic emotions using a 7-point scale. After scoring by pressing a button, the participants entered the next trial. In competitive and cooperative situations, the three experimental conditions were (a) positive or negative academic emotion story—watching, (b) positive or negative academic emotion story—emotional suppression, and (c) positive or negative academic emotion story—emotional expression. Ten stories of academic emotion constituted each experimental condition (five positive stories of academic emotion and five negative stories); they were balanced under the three conditions. In addition, considering that the academic emotional stories and regulation strategies in the previous trial were likely to have an impact on the academic emotional regulation in the later trial, in Study 1 we used 10 trials under the same experimental conditions. The entire experiment lasted about 25 min.

Before the formal experiment the experimenter informed the participants of the entire process and explained the definitions and examples of strategies for regulating academic emotion (watching, emotional suppression, and emotional expression) to be used by the participants to ensure that they understood the relevant contents of the experiment. Two points require attention: First, in the competitive situation, participants were told that the object of emotional suppression and expression is other students in the competitive relationship; in the cooperative situation, participants used emotional suppression and expression against the classmates who cooperated with them to complete the task. Second, to familiarize participants with the experimental process and the strategies used for regulating the academic emotion, they were asked to complete six additional trials (two under each strategy) as practice before the formal experiment. During the practice, participants were required to verbally report how they used the strategies for regulating emotion presented on the screen to control the academic emotion induced by the story to ensure that they could accurately regulate their academic emotion in strict accordance with the requirements of the guidance during the experiment. After the practice the formal experiment began (the materials used in the practice experiment were not used in the formal experiment). After the formal experiment each participant was given a neutral pen or notebook as a reward.

#### 2.1.5 Analytic strategy

The data from E-prime 2.0 was analyzed using the statistical software package SPSS 20. The initial statistical procedure involved conducting repeated measures ANOVA to examine situational differences in the associations between positive academic emotion and the strategies for expressing them. Subsequently, situational differences between those strategies and negative academic emotion were also analyzed using repeated measure ANOVA.

### 2.2 Results

First, the situation and strategy for expressing academic emotion were used as independent variables, and the intensity of positive academic emotion was used as the dependent variable for repeated measurement ANOVA (see Table 1). Mauchly’ test of sphericity revealed that the data satisfied the spherical hypothesis (*p* = 0.13 > 0.05). The results showed that the main effect of the situation was not significant, but the main effect of the strategy for expressing academic emotion was significant, *F* (2, 126) = 14.60, *p* < 0.001, *η*_p_^2^ = 0.19. *Post hoc* tests showed that compared with the watching condition (*M* = 5.52, *SD* = 0.97) and the emotional expression condition (*M* = 5.55, *SD* = 1.08), adolescents reported a lower intensity of positive academic emotions when suppressing emotion (*M* = 4.93, *SD* = 1.10). No significant difference emerged in the intensity of positive academic emotion between watching and expressing. The interaction between the situation and strategies for expressing academic emotion was not significant.

**Table 1:** The intensity of academic emotions under different strategies for expressing academic emotions.

	Positive academic emotions (*M* ± *SD*)	Negative academic emotions (*M* ± *SD*)
Watching	5.52 ± 0.97	4.16 ± 0.95
Emotional suppression	4.93 ± 1.10	4.00 ± 1.12
Emotional expression	5.55 ± 1.08	3.97 ± 0.99

Second, we computed repeated measure ANOVA on the intensity of negative academic emotion with the situation and strategy for expressing academic emotion as predictors. Mauchly’ test of sphericity revealed that the data satisfied the spherical hypothesis (*p* = 0.85 > 0.05). We found that the main effect of the situation was not significant. The main effect of the strategy for expressing academic emotion was significant, *F* (2, 126) = 3.13, *p* < 0.05, η_p_^2^ = 0.05. *Post hoc* tests showed that adolescents reported lower levels of negative academic emotional intensity when expressing emotion (*M* = 3.97, *SD* = 0.99) compared to the watching condition (*M* = 4.16, *SD* = 0.95). No significant difference emerged between the emotional suppression condition (*M* = 4.00, *SD* = 1.12) and the other two conditions. The interaction between the situation and the strategy for expressing academic emotion was not significant.

### 2.3 Discussion

Results from Study 1 reveal that the relations between academic emotions and strategies to express them have situational stability. That is, when adolescents experience positive academic emotions in both competitive and cooperative situations, emotional suppression can weaken positive academic emotions compared with watching conditions and expression of emotion; but no significant difference emerged in the intensity of positive academic emotions in the conditions of watching and emotional expression. When adolescents experienced negative academic emotions, no significant difference appeared in the intensity of negative academic emotions between emotional suppression and watching conditions; but compared with the watching condition, emotional expression can reduce the intensity of negative academic emotions.

First, partially consistent with the hypothesis b of Study 1, and consistent with the results of previous studies (Gross and Levenson, 1997; Schall et al., 2016), our findings reveal that suppression of negative emotions did not change their intensity, but suppression of positive emotions weakened their intensity. Thus, hiding an individual’s emotions did not lead to a better emotional experience. So why did emotional suppression reduce the subjective positive emotional experience rather than the negative emotional experience? One possible explanation is that people tend to control their negative emotions more often than their positive ones (Wallbott and Scherer, 1989). Therefore, individuals’ experience of negative emotions and emotional suppression are more likely to be disconnected than positive emotions, which may have led them to rely less on their expressive behavior when evaluating their experiences of negative emotions (Gross and Levenson, 1997).

Second, inconsistent with the hypothesis a of Study 1, results from Study 1 demonstrate that the expression of positive academic emotion did not change it, but the expression of negative academic emotion weakened it. This may be so because expressing negative emotions to others is a means of alleviating distress (Stanton et al., 2000), which can diminish the frequency of intrusive thoughts about stressful events and reduce the individual’s sense of helplessness concerning negative emotions (Kennedy-Moore and Watson, 2001). Meanwhile, putting negative emotional experiences into words can also help individuals to recognize, understand, interpret, and accept their inner subjective negative emotional state (Kennedy-Moore and Watson, 2001).

## 3 Study 2

In Study 2 we examined the relationship between strategies for expressing academic emotion and peer acceptance in competitive and cooperative situations.

### 3.1 Method

#### 3.1.1 Participants

A total of 113 adolescents from Grades 7 through 12 (age range = 12–18 years; *M*_age_ = 15.34 years, *SD* = 1.62 years; 46 boys) in Shandong province in China were randomly selected to participate in this study. All adolescents had normal or corrected vision, with no color blindness or color weakness. Over 90% of them were of Han nationality, the dominant ethnic group in China. Sensitivity analyses indicated our sample size to be sufficiently powered at 80% for detecting small effects in regression models (Cohen’s *f* = 0.13).

#### 3.1.2 Measures

As for the assessment of the target classmate’s acceptance level, in Study 2 we set a hypothetical situation and asked participants to evaluate their acceptance of the target classmate who formed competitive or cooperative relationships with them in a particular situation. The experiment was divided into two parts: First, the experimenter described the situation the participants would encounter, which included the target classmate who (a) formed a competitive or cooperative relationship with them and (b) suppressed or expressed positive or negative academic emotions in their presence. For example, “Imagine now that you encounter a situation: In the process of cooperative learning, the target classmate you are working with has completed the learning task and has experienced very positive academic emotions (e.g., happiness, pride); he or she has suppressed positive academic emotions in your presence. For example, he or she does not show you the happiness felt at the moment.” The participants were then asked to rate their acceptance of the target classmate.

##### 3.1.2.1 Acceptance

We assessed the acceptance of the target individual expressing or suppressing emotion using three items (e.g., “I would like to have this classmate as a coworker,” rated on a 7-point scale) derived from the Acceptance Questionnaire (Schall et al., 2016; originally developed by Wentzel, 1994). A total score was computed by averaging the items, with higher values indicating higher acceptance. In the competitive and cooperative situations, Cronbach’s α for this questionnaire was between 0.92 and 0.96 under the conditions of the suppression or expression of positive or negative academic emotions. The results of eight CFA analyses indicated that all eight models were saturated.

#### 3.1.3 Procedure

The study was first approved by the institutional review board at our university. Written consents were then obtained from all adolescents and their parents through the schools. All of the measures were tested by a team of trained psychology postgraduate students in a regular classroom. These testing sessions took approximately 10 min.

#### 3.1.4 Analytic strategy

The statistical software package SPSS 20 was utilized for data analysis. Initially, ANOVA was employed to examine the relationship between emotional suppression and peer acceptance in competitive and cooperative situations. Subsequently, the associations between emotional expression and peer acceptance in competitive and cooperative situations was also assessed using ANOVA. Finally, a paired sample t-test was conducted to investigate the difference in acceptance after the target individuals suppressed or expressed positive or negative academic emotion in the two situations.

### 3.2 Results

First, we examined the impact of Situation × Academic emotion type on acceptance for suppressing emotion. The results of the ANOVA on acceptance (Mauchly’ test of sphericity yielded a significant result, *p* < 0.001, and the *F* test was adjusted using the Greenhouse–Geisser) showed a nonsignificant main effect of the situation and a significant main effect of academic emotion type, *F* (1, 112) = 25.66, *p* < 0.001, *η*_p_^2^ = 0.19. This effect was qualified by a significant Situation × Academic emotion type interaction, *F* (1, 112) = 9.81, *p* < 0.01, *η*_p_^2^ = 0.08. Further analysis showed that for the suppression of positive academic emotion, acceptance in the competitive situation (*M* = 4.40, *SD* = 1.55) was significantly higher than that in the cooperative situation (*M* = 3.89, *SD* = 1.38), *F* (1, 112) = 10.63, *p* < 0.01. In addition, participants in the competitive situation evaluated the target student as more acceptable when this student suppressed negative emotions (*M* = 4.74, *SD* = 1.32) than when she or he suppressed positive emotions (*M* = 4.40, *SD* = 1.55), *F* (1, 112) = 3.92, *p* < 0.05, in the cooperative situation, the acceptance of the target individual who suppressed negative academic emotions (*M* = 4.84, *SD* = 1.32) is significantly higher than that of the target individual who suppressed positive academic emotions (*M* = 3.89, *SD* = 1.38), *F* (1, 112) = 39.62, *p* < 0.001.

Second, we examined the impact of Situation × Academic emotion type on acceptance of expressing emotion. An ANOVA on acceptance (Mauchly’ test of sphericity yielded a significant result, *p* < 0.001, and the *F* test was adjusted using the Greenhouse–Geisser) revealed (a) a significant main effect of situation, (b) a significant main effect of the expression of emotion, and (c) a significant Situation × Academic emotion type interaction. The results showed that for the expression of positive academic emotion, acceptance in the competitive situation (*M* = 4.61, *SD* = 1.54) was significantly lower than that in the cooperative situation (*M* = 5.23, *SD* = 1.27), *F* (1, 112) = 16.55, *p* < 0.001. Moreover, in both competitive and cooperative situations, participants accepted the target student significantly less when this student expressed negative emotions (*M* = 3.97, *SD* = 1.42; *M* = 4.07, *SD* = 1.40) than when he or she expressed positive emotions (*M* = 4.61, *SD* = 1.54; *M* = 5.23, *SD* = 1.27), *F* (1, 112) = 13.40, *p* < 0.001, *F* (1, 112) = 57.48, *p* < 0.001.

Third, we computed a paired sample *t*-test to investigate the difference in acceptance after the target individuals suppressed or expressed positive or negative academic emotion in the two situations. The results (see Table 2) indicated that in the competitive situation no significant difference appeared in acceptance following the suppression and expression of emotion when the target individuals experienced positive academic emotion (*M* = 4.40, *SD* = 1.55; *M* = 4.61, *SD* = 1.54). When experiencing negative academic emotion, the acceptance of the target individual suppressing emotion (*M* = 4.74, *SD* = 1.32) was significantly higher than when expressing emotion (*M* = 3.97, *SD* = 1.42), *t* (112) = 4.52, *p* < 0.001, Cohen’s *d* = 0.56. In the cooperative situation when the adolescents experienced positive academic emotions, the acceptance of the target individual suppressing emotion (*M* = 3.89, *SD* = 1.38) was significantly lower than when expressing emotion (*M* = 5.23, *SD* = 1.27), *t* (112) = −7.63, *p* < 0.001, Cohen’s *d* = 1.01; and when experiencing negative academic emotions, acceptance of the target individual when suppressing emotion (*M* = 4.84, *SD* = 1.32) was significantly higher than that when expressing emotion (*M* = 4.07, *SD* = 1.40), *t* (112) = 4.72, *p* < 0.001, Cohen’s *d* = 0.57.

**Table 2:** Acceptance under the conditions of the suppression or expression of positive or negative academic emotions.

	Positive academic emotions (*M* ± *SD*)		Negative academic emotions (*M* ± *SD*)	
	Emotional suppression	Emotional expression	Emotional suppression	Emotional expression
Competitive situation	4.40 ± 1.55	4.61 ± 1.54	4.74 ± 1.32	3.97 ± 1.42
Cooperative situation	3.89 ± 1.38	5.23 ± 1.27	4.84 ± 1.32	4.07 ± 1.40

### 3.3 Discussion

Consistent with the viewpoints of emotional regulation flexibility (Bonanno and Burton, 2013; Aldao et al., 2015) and self-regulated learning theory (Boekaerts, 2007; García-Pérez et al., 2021), the results of the relationship between strategies for expressing academic emotion and acceptance in Study 2 emphasize the role played by the situation, that is, compared with the cooperative situation, the suppression of positive academic emotion in the competitive situation garnered a higher level of peer acceptance, and expression of positive academic emotion garnered a lower level of peer acceptance. This may be related to the rivalry of competitive situations. Based on the theory of social interdependence (Johnson and Johnson, 1998), competitive situations make individuals experience out-group membership, and the interdependence of cooperative situations that makes individuals experience in-group membership (Liu and Wang, 2005; Paulus et al., 2016). Individuals who suppress the positive emotions they experience in competitive situations may be perceived as unpretentious and protective of others’ feelings (Chiang, 2012). If positive academic emotions are suppressed in a cooperative situation, individuals may be considered not to have integrated into the cooperative group and to have a sense of distance from the group members. On the contrary, if individuals express the positive academic emotions they experience in competitive situations, doing so may disrupt the social relations characterized by self-interest and confrontation in such situations (Greenaway and Kalokerinos, 2017). Because such behavior not only indicates that the individual has harmed the interests of the competitor (Van Kleef et al., 2010) but also that the individual is showing off, causing the others to feel a sense of Schadenfreude (Cikara et al., 2014), the conveyed sense of superiority may cause the discomfort of the competitor (Chiang, 2012). However, expressing one’s positive academic emotions in a cooperative situation may be a sign of trust in in-group members. By sending positive information, such as happiness or recognition of their efforts to group members (Paulus et al., 2016), the communication among them will be smoother and the cooperation will be more efficient (Van Kleef, 2010). Thus, when individuals experience positive academic emotions, being “happy alone” is more suitable in a competitive situation, but being “happy together” is more suitable in a cooperative situation.

The results from Study 2 reveal that in the competitive situation, no significant difference occurred between the peer acceptance level of adolescents who suppress positive academic emotion and those who express emotion. One possible explanation is that the effects of suppressing and expressing positive academic emotions are two sides of the same coin. Another possibility is that Study 2 does not break down the competition situation further. Individuals may be characterized as more likable and amiable when they express rather than suppress positive emotions in the nonoutperformance situation (Schall et al., 2016). Therefore, future research must involve further subdivision of the competition situation for more in-depth discussion and analysis.

Notably, partially consistent with the hypothesis a of Study 2, in the cooperative situation the peer acceptance level of the suppression of positive academic emotion was significantly lower than that of expression. As stated in the theory of social interdependence, in the context of cooperation, the relationship between members involves cooperation, interdependence, and support of one another. The emotional states of the group members may affect the emotions of other members (Podsakoff et al., 2000). The suppression of positive academic emotions may be equated with distancing from group members while the expression of positive academic emotions is more compatible with the cooperative situation (Van Kleef et al., 2004). The information conveyed may enhance the pleasant experience of group members, ensure the smooth progress of cooperation, and drive the further improvement of team performance (Barsade, 2002; Van Kleef et al., 2010).

In addition, Study 2 also shows that in both competitive and cooperative situations, the peer acceptance level for the target individual suppressing negative academic emotion is significantly higher than that of the one expressing the emotion. This means that suppression, not expression, of negative academic emotions may be more adaptive and more conducive to achieving the emotional regulation goal of good interpersonal relationships in both competitive and cooperative situations. Although some researchers have found that the expression of negative emotions adapted to the needs of the situation may signal trust and popularity and may, therefore, initiate a new relationship (Graham et al., 2008), Study 2 shows inconsistent results. This may be so because in the academic environment, no matter what kind of learning situation they are in, the ultimate goal of individuals is to achieve better academic development. If the way others express their academic emotions interferes with or threatens their ultimate learning goals, individuals will not judge them more positively. Especially when others express negative emotions, the negative energy transmitted by them will worsen interpersonal effects (Van Kleef, 2010). For example, expressing anger usually implies that the other person’s views or behaviors are unacceptable to the expresser intending to change the behavior (Fischer and Manstead, 2008). Especially in group situations the expression of anger is often accompanied by social exclusion, which will increase the social distance among group members and reduce the sense of belonging (Heerdink et al., 2013). At this point suppressing inappropriate emotions may be a more appropriate choice (Cole et al., 2008). Group members may perceive other members’ emotional suppression as “faking in good faith,” representing a well-intended means of promoting good interpersonal interaction (Hu and Shi, 2015).

## 4 General discussion: implications, limitations, and future directions

By examining the relationship between strategies for expressing academic emotions and the emotions themselves and peer acceptance of adolescents in competitive and cooperative situations, we answer not only questions about the relationship but also the similarities and differences between the two related models. That is, the relationships between academic emotions and strategies for expressing them have situational stability while the relationships between the strategies and peer acceptance have situational specificity. In addition, from the perspectives of their own academic emotional experience and peer acceptance, this study makes clear which strategies for expressing academic emotional offer more adaptive value for individuals in different situations: In competitive and cooperative situations when experiencing positive academic emotions, emotional expression may be more adaptive, and this adaptability may be more obvious in cooperative situations. When experiencing negative academic emotions, emotional expression is more adaptive in terms of their own academic emotional experience; and the use of the emotional suppression is more adaptive in terms of peer acceptance.

These findings not only validate the applicability of emotional regulation flexibility in the academic environment and enrich and expand relevant theoretical and empirical studies (e.g., self-regulated learning theory) on strategies for expressing emotion but also encourage educators and parents to realize the importance of adolescents using adaptive strategies for emotive expression in different situations. Furthermore, the findings can help them to take appropriate measures to guide adolescents to use strategies for expressing emotion flexibly according to the situational requirements. For example, teachers can embed knowledge of strategies for expressing academic emotions in their teaching process, and educators and parents can also encourage adolescents to express their positive academic emotions in competitive situations, especially in cooperative situations, and to express their negative emotions when they are unhappy but want to be happy; doing so is conducive to the ultimate realization of promoting the better development of adolescents. These findings also have longer-term benefits for adolescents, for example, helping to guide them in adopting more appropriate emotional regulation strategies in their future college and work lives to promote their healthy development.

Notwithstanding, some limitations of the present study should be considered. First, for adolescents an obvious feature is the increasingly refined management of emotional expression (Zeman et al., 2006). When experiencing positive and negative emotions, adolescents may use a variety of strategies for regulating emotion and produce a range of results accordingly (Webb et al., 2016); therefore, future research should involve the exploration of specific emotions to clarify the effect of using strategies for expressing emotion on a single emotion (e.g., anxiety). Second, academic emotions are domain-specific (Xu and Gong, 2009), so academic emotional experiences may differ for students in various disciplines. For example, math anxiety is common among students in math subjects (Si et al., 2022); therefore, future research should focus on specific disciplines to explore the impact of strategies for expressing emotion on individual learning and other aspects. Third, given that the average arousal level of positive academic emotions is higher than that of negative academic emotions, which could potentially impact the internal validity of the Study 1, it is advisable to consider employing more representative experimental material for further in-depth investigation in future studies. Fourth, the experiment is set to be a hypothetical situation in Study 2. Because the target classmate is imagined, and the outcome may differ from that of the specified classmate; therefore, relevant studies may be supplemented and sample sizes expanded in the future to validate these results. Finally, Study 2 involves only the relationship between strategies for expressing academic emotion and peer acceptance, but the social outcomes include more than that. Previous studies have also shown that strategies for expressing emotion are closely related to social support, relationship satisfaction, and other aspects (Velotti et al., 2016; Tsai et al., 2017). In addition, more forms of emotion regulation are related to academic settings (e.g., cognitive reappraisal, Somerville and Whitebread, 2019). Carrying out related exploration in the academic field is necessary to enrich the research on this topic.

## Data availability statement

The original contributions presented in the study are included in the article/supplementary material, further inquiries can be directed to the corresponding author.

## Ethics statement

The studies involving humans were approved by East China Normal University Committee on Human Research Protection/East China Normal University. The studies were conducted in accordance with the local legislation and institutional requirements. Written informed consent for participation in this study was provided by the participants’ legal guardians/next of kin.

## Author contributions

YL: Writing – review & editing, Writing – original draft, Investigation, Formal analysis, Conceptualization. XC: Writing – review & editing, Writing – original draft. BS: Writing – review & editing, Writing – original draft, Conceptualization. SZ: Writing – review & editing, Conceptualization.
